# Fault Diagnosis of Lubrication Decay in Reaction Wheels Using Temperature Estimation and Forecasting via Enhanced Adaptive Particle Filter

**DOI:** 10.3390/s23031474

**Published:** 2023-01-28

**Authors:** Mahdi Alidadi, Afshin Rahimi

**Affiliations:** Department of Mechanical, Automotive and Materials Engineering, University of Windsor, 401 Sunset Ave, Windsor, ON N9B 3P4, Canada

**Keywords:** fault diagnosis, fault prognosis, fault estimation, particle filters, reaction wheels, lubrication decay, remaining useful life

## Abstract

Reaction wheels (RW), the most common attitude control systems in satellites, are highly prone to failure. A satellite needs to be oriented in a particular direction to maneuver and accomplish its mission goals; losing the reaction wheel can lead to a complete or partial mission failure. Therefore, estimating the remaining useful life (RUL) over long and short spans can be extremely valuable. The short-period prediction allows the satellite’s operator to manage and prioritize mission tasks based on the RUL and increases the chances of a total mission failure becoming a partial one. Studies show that lack of proper bearing lubrication and uneven frictional torque distribution, which lead to variation in motor torque, are the leading causes of failure in RWs. Hence, this study aims to develop a three-step prognostic method for long-term RUL estimation of RWs based on the remaining lubricant for the bearing unit and a potential fault in the supplementary lubrication system. In the first step of this method, the temperature of the lubricants is estimated as the non-measurable state of the system using a proposed adjusted particle filter (APF) with angular velocity and motor current of RW as the available measurements. In the second step, the estimated lubricant’s temperature and amount of injected lubrication in the bearing, along with the lubrication degradation model, are fed to a two-step particle filter (PF) for online model parameter estimation. In the last step, the performance of the proposed prognostics method is evaluated by predicting the RW’s RUL under two fault scenarios, including excessive loss of lubrication and insufficient injection of lubrication. The results show promising performance for the proposed scheme, with accuracy in estimation of the degradation model’s parameters around 2–3% of root mean squared percentage error (RMSPE) and prediction of RUL around 0.1–4% error.

## 1. Introduction

Over the past four decades, condition monitoring and, in particular, fault diagnosis and prognosis (FDP) for condition-based maintenance (CBM) and safety assurance have been fields of interest [1,2]. The primary purpose of FDP and CBM is to detect, isolate, and predict the propagation of incipient faults in their early stages. The use of FDP in field research has been widespread, ranging from manufacturing to aerospace. However, Li-ion batteries and rotating machinery systems for electrical and industrial applications have received the most attention.

In aerospace, the low cost of developing and launching modular and nano/microsatellites has contributed to their rapid growth in the past few years. These satellites are usually used for educational or monitoring purposes [3] and have a long-expected lifetime. In general, systems are prone to faults and failures during their lifetime. Moreover, the small satellite’s housing space limitations make carrying spare parts and incorporating hardware redundancy units virtually impractical. These reasons make fault-tolerant controllers (FTC) and analytical redundancy algorithms crucial.

The attitude control system (ACS), as one of the essential parts of a satellite, is prone to faults [4], and its failure has been the main reason for many satellite mission failures. Both long-term and short-term estimates of RW’s RUL can be beneficial. The satellite’s operators can remedy mission failure using the knowledge of RUL predictions for prioritizing mission tasks. Additionally, RUL prediction over extended periods can help the operators develop alternative attitude control methods to avoid potential future collisions or failures. Kepler is an example of substituting control approaches after losing its first and second RWs [5]. Table 1 lists more examples of RWs and ACS failures.

In the past decades, many researchers have worked on determining and potentially extending the lifespan of these critical satellite components, namely, RWs [7]. Creating an effective prognosis system is particularly challenging due to the complexity of faults, the lack of knowledge about how they progress, and the inherent uncertainty in predicting future failure or degradation [5]. Therefore, despite considerable research in fault detection, diagnostics, and isolation (FDDI) of ACS and RW satellites, few are on these systems’ failure prognosis and RUL estimation.

A combination of a backpropagation neural network with a similitude method is presented in [8] for RUL estimation of momentum wheels (MW). This method relies on temperature and angular speed as indicators of bearing friction torque and critical parameters.

Another data-based method using a long short-term memory (LSTM) neural network is proposed in [9] to estimate the motor torque of RWs as the health index (HI). In the first step of this study, an LSTM is trained using historical data to forecast RWs’ angular speed ($\omega_{m}$) and motor current ($I_{m}$). The $\omega_{m}$ and $I_{m}$ are then fed into another LSTM to estimate the RW torque as its HI. In [10], the authors extend their work by suggesting a “Three-stage Data-Driven Approach” for estimating RWs’ RUL. The third step estimates the RUL based on a pre-defined failure threshold for the estimated HI.

Muthusamy and Kumar [11] present a data-driven RUL estimation approach for a single gimballed control moment gyros (CMG) with output residuals as HI for the CMG. The polynomial general path model (GPM) is applied for the prognostic method. Moreover, the Bayesian updating method is employed to combine real-time and historical run-to-failure data for more accurate estimation from the GPM. The advantage of this method is that it only requires attitude rate measurements to work; no other measurements are required for this method.

The accuracy of all the reviewed data-driven and hybrid methods is highly dependent on the amount of labeled run-to-failure data sets from the same type of system and under the same operating conditions [12], both of which are difficult and expensive to acquire in aerospace systems. This shortcoming of data-driven and hybrid methodologies highlights the need for model-based methods.

The model-based prognostics methods are generally associated with an estimation method for the desired system states. The Kalman filter (KF) is one of the most widely used stochastic estimation methods [13]. KF-based prognosis has the advantage of low computational burden as it does not require all the previous measurements to update the most recent state, but rather only the most recent measurements to do so. However, some limitations restrict these methods, including their inability to handle nonlinear models and non-Gaussian noise. Yun et al. [14] use a multi-scale extended Kalman filter (EKF) for RUL estimation of RWs’ motors. As the EKF method uses local linearization about the estimated state, it can be used with nonlinear systems. The damping coefficient is estimated using micro EKF, input current, and measured output angular velocity in the first stage of this method. Using a macro EKF to adjust the degradation model, the system’s RUL is estimated via the estimated damping coefficient as the motor’s HI. Based on the estimated states of the system and their distributions, Monte Carlo (MC) simulations are conducted until the system’s HI reaches a pre-defined threshold and RUL’s probability density function (PDF) can be computed. Although this method performs well for the short-term prognosis of state-of-health (SOH), it lacks accuracy for long-term diagnosis and RUL prediction.

Despite EKF’s ability to estimate parameters of nonlinear degradation models, errors in Jacobian matrices with higher-order terms can majorly affect EKF’s accuracy [15]. Rahimi et al. [16] present a model-based FDDI method using a covariance-based adaptive unscented Kalman filter (AUKF) to estimate and track sudden changes in a system’s non-measurable parameters. They estimate the motor torque and bus voltage of Ithaco Type A RWs as unknown system parameters, utilizing $\omega_{m}$ $I_{m}$ as measurement. Later, in [17], Rahimi et al. present a RW’s RUL estimation method using estimated motor torque as the system’s HI. For this purpose, a PF is used for online parameter tracking of the HI’s exponential deterioration trend. The simulation results show that this method can estimate the RUL of the RW while handling both Gaussian and non-Gaussian noise. Bialke et al. [6,18] conducted a root cause analysis of Ithaco RWs failure and found a strong correlation between geomagnetic storms and these failures. These articles indicate that the common fault in RWs’ friction factors is abrupt changes in dry friction, which can cause short-term failure or be resolved by high rotation speed. Considering these findings, the changes in the wet friction factor are fewer than those in dry friction [6], which would not be a perfect choice as an incipient fault that causes long-term failure. In most of the published literature for fault prognosis of space systems and actuators, including RWs, the focus has been on estimating torque/back-electro motive force gains or bus voltage to identify anomalies in system behavior. The effects of lubrication and temperature changes in the RW system have rarely been looked at and investigated.

**Figure 1:**
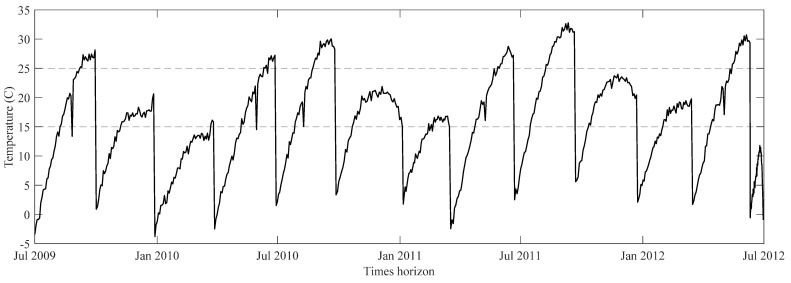
The seasonal thermal trend of Kepler spacecraft’s RW.

As a rotary system, the bearing unit of an RW requires proper lubrication for its entire operational life. Typically, the lubricant stored in the bearing housing is sufficient for 3–4 years of operation under normal conditions, and adding a supplementary lubrication system will extend the life of the system [6]. However, the limited amount of lubricant that a nano/micro-satellite can carry and its harsh operating environment make it impossible to recharge lubrication reservoirs. Therefore, a HI based on the cumulative consumption of lubricant for the bearing unit of MWs is proposed in [19]. As a first step, the authors use the physics of failure to develop a degradation model for lubrication loss. Lubrication evaporation, degradation, and migration, driven by the vacuum, thermal gradient, and centrifugal force, are the primary sources of lubrication loss in the bearing unit of an RW. The experiments in [19,20,21] also show that, among the three primary sources of lubricant loss, lubrication evaporation and creeping play the most critical roles, and they are both functions of lubricant temperature. The analysis of the main causes of lubrication loss led to the development of a nonlinear temperature function as a degradation model. Using maximum likelihood and experimental data, the authors were able to estimate the parameters of the proposed degradation model. Applying empirical mode decomposition (EMD) to the measured temperature from the experimental setup, a trend was obtained in the lubricant’s temperature. Next, the obtained trend is fed into the degradation model to estimate future lubrication consumption. As a means of dealing with uncertainty, a bootstrap simulation is also used in this study. While this method provides a long-term RUL estimation for MW, it only considers monotonic temperature trends and ignores the effect of temperature fluctuations caused by the satellite’s orientation relative to the sun. Figure 1 shows a sample of the seasonal thermal trend for the Kepler space telescope’s RW [22].

In [23], the authors proposed a fast and accurate end-to-end architecture for detecting and identifying the anomalies occurring in spacecraft RWs using a One-Dimensional Convolutional Neural Network (1D-CNN) with an LSTM network architecture. The types of faults they investigated in their work include low and high bus voltage faults, motor current loss, and high friction faults. Although they investigated a friction-related fault in the system, they did not explicitly investigate the temperature and lubrication effects on system performance and its RUL. Zhang et al. [24] aimed to design a prediction framework that meets satellite state on-orbit prediction requirements. These requirements include data reconstruction, similar data screening, self-revised prediction, and the fuzzy expression of results. They proposed a Fourier-based Broad Learning System (BLS) to improve the depiction accuracy of a standard BLS in learning satellite telemetry data that requires less computational and time resources. The metric they were interested in determining through their proposed data-driven approach was the state of health, which was available in the telemetry data they started their work with. Therefore, the notion of individual physical and tangible satellite or RW parameters was not in their work scope. From the reviewed literature, while active and passive lubrication injection systems have been proposed to ensure proper bearing lubrication in satellite missions lasting 30 years or longer, the estimation of RUL based on the remaining lubrication and failure prognostic due to fault in the injection system remains a challenge. The existing literature does not explicitly address the investigation of RW RUL prediction based on temperature or lubrication anomalies in RWs. Therefore, this study aims to address this gap in the literature.

**Figure 2:**
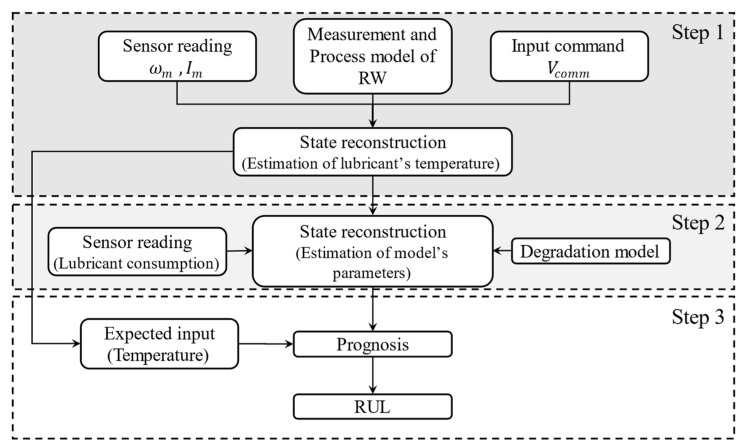
The steps to forecast the RUL of an RW.

As shown in Figure 2, to address these problems, we propose a three-step prognosis approach for RUL estimation of RWs based on the lubrication consumption of the bearing unit as follows:

**Step 1**. Since direct measurement of the lubricant’s temperature under the operation condition is not practical, in the first step of this research, we investigate the estimation of the lubricant temperature using angular velocity ($\omega_{m}$) and motor current ($I_{m}$) as measurements and a high-fidelity model of the Ithaco RW proposed in [25,26].

**Step 2**. In the second step, an online parameter tracking of the lubricant consumption model, as well as the degradation model, is conducted using the estimated lubricant’s temperature and the measured amount of injected lubricant.

**Step 3**. The last step is to predict the RUL of RW based on two scenarios of failure: (1) The cumulative consumption of lubricant reaches the amount stored in the system. (2) Deficiencies in lubrication injection and supplemental lubrication systems lead to bearing unit dry-out. In the first scenario, the fault occurs during normal operating conditions and will be accelerated by lubrication bleeding or RW heating. However, a fault in the supplementary lubrication system causes an unbalanced injection of lubrication in the second scenario (see Figure 2).

In addressing the identified gap in the literature, this study offers the following novelties:

(1) Investigation of the temperature and lubrication effects on RWs deterioration and its impact on RWs remaining useful life that has not been previously addressed in the literature;
(2) A three-step process to determine the RUL of RWs undergoing temperature and lubrication malfunctions that include a novel particle filter resampling approach to improve the accuracy and efficiency of estimation results.

The remainder of this paper is organized as follows: In Section 2, preliminaries are provided to familiarize the reader with the background contents. In Section 3, the methodology used in this study is detailed. In Section 4, results and discussions of this study’s outcomes are provided, and Section 5 provides concluding remarks and future directions for this study.

## 2. Preliminaries

This section provides background and theories used in this study to help the reader follow the content easier and for the paper to be self-contained. This includes satellite RW modeling, degradation modeling, and the state estimation theory used in this work.

### 2.1. Satellite’s RW Model

**Figure 3:**
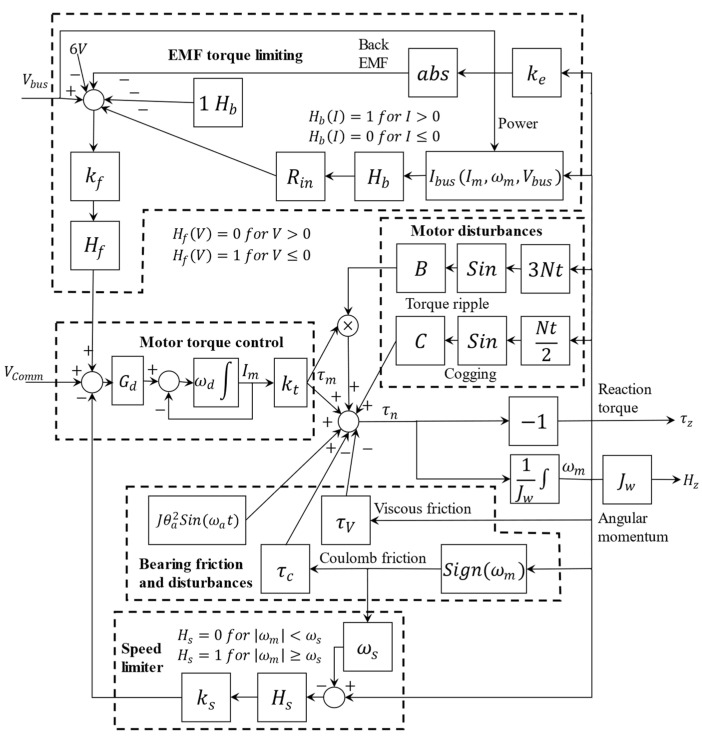
High-fidelity RW dynamic adapted from [25] where $k_{t}$ feeds to multiplication term in Bsin(3Nt) and uses a low-pass filter in the motor torque control block.

The two primary sources of lubrication loss in RW’s bearing unit are evaporation and creeping. They are both functions of the lubricant’s temperature. As a result, monitoring the temperature is crucial to developing an HI based on cumulative lubrication loss. It is generally not possible to directly measure the lubricant’s temperature. Therefore, in this study, an estimation approach is employed that uses angular velocity ($\omega_{m}$) and motor current ($I_{m}$), a high-fidelity model of RW, and $V_{comm}$ as the input of the system. A model of Ithaco ’type A’ RW by Goodrich [25] is adapted and used for this purpose, as shown in Figure 3, with its model parameters listed in Table 2.

Now that the RW model is fully detailed, modeling the degradation trend for the analysis in this study is explained in the next section.

Several essential loops in the RW model ensure precision. Figure 3 shows these loops with dashed polygons, and they can be mathematically expressed as:

1. The electro-motive force (EMF) torque limiting loop:
  (1) $$I_{bus}=\left(\frac{1}{V_{bus}-1}\right)\left(I_{m}^{2}R_{B}+0.04\left|I_{m}\right|V_{bus}+P_{q}+\omega_{m}I_{m}K_{e}\right)$$
  where $V_{bus}$ and $I_{bus}$ stand for bus voltage and current, respectively, $K_{e}$ is the back electro-motive force (BEMF) voltage gain, and $\omega_{m}$ represents wheel’s angular speed;
2. The Coulomb friction is created by the dry friction within bearings. This loss torque is independent of the angular velocity or the temperature;
3. The negative feedback viscous friction:
  (2) $$\tau_{v}=\left(0.0049-0.0002\left(T_{lub}+30\right)\right)\omega_{m}$$
  where $\tau_{v}$ and $T_{lub}$ are the viscous torque and the lubricant’s temperature, respectively. This friction is driven by the resistive force between the bearing surface in relative motion through the lubricant and is in addition to the Coulomb friction;
4. The negative feedback speed limiter loop prevents the wheel from accelerating to unsafe speeds. This loop is triggered by exceeding the speed threshold, $\omega_{s}$, and generates negative feedback via gain $K_{s}$, which feeds the torque command;
5. The motor torque control unit is a voltage-controlled current source with a gain $G_{d}$. Through a constant torque gain $K_{t}$, the motor delivers a torque proportional to the current driver, $I_{m}$. Moreover, a non-ideal RW has a limitation for following the input frequency, which can be presented as a low-pass filter with a bandwidth of 9 rad/sec, $\omega_{d}$;
6. $\tau_{noise}$ is an extremely low-frequency torque variation forced by lubricant dynamics and can be presented as follows:
  (3) $$\tau_{noise}=J-w\;\theta_{a}\omega_{a}^{2}\sin\left(\omega_{a}t\right)$$

The nonlinear mathematical model of the RW in Figure 3 can be expressed as follows:

(4) $${\dot{I}}_{m}=G_{d}\omega_{d}\left[f_{3}\left(\omega_{m},I_{m}\right)-f_{5}\left(\omega_{m}\right)\right]-\omega_{d}I_{m}+G_{d}\omega_{d}V_{Comm}\;{\dot{\omega }}_{m}=\frac{1}{J_{w}}\left\{f_{1}\left(\omega_{m}\right)+k_{t}I_{m}\left[f_{2}\left(\omega_{m}\right)+1\right]-\tau_{v}\omega_{m}-\tau_{c}f_{4}\left(\omega_{m}\right)+\tau_{noise}\right\}$$

where

(5) $$f_{1}\left(\omega_{m}\right)=Csin\left(\frac{Nt}{2}\omega_{m}\right)\;f_{2}\left(\omega_{m}\right)=Bsin\left(3Nt\omega_{m}\right)\;f_{3}\left(\omega ,I_{m},V_{bus}\right)=H_{f}\left(V\left(\omega_{m},I_{m},V_{bus}\right)\right)V\left(\omega_{m},I_{m},V_{bus}\right)\;f_{4}\left(\omega_{m}\right)=sign\left(\omega_{m}\right)\;f_{5}\left(\omega_{m}\right)=K_{s}\left[\left|\omega_{m}\right|-\omega_{s}\right]H_{s}\left(\left|\omega_{m}\right|-\omega_{s}\right)\;V\left(\omega_{m},I_{m},V_{bus}\right)=K_{f}\left[V_{bus}-6-\left[1+R_{in}I_{bus}\right]H_{b}\left(I_{bus}\right)-\left|K_{e}\omega_{m}\right|\right]$$

The $f_{1}$ to $f_{5}$ terms indicate different building blocks of the RWs while $I_{m}$ and $V_{comm}$ are the motor current and the torque command voltage, respectively.

For the numerical solution of the nonlinear model, the discontinuous functions are needed to be approximated. In [25], an approximation based on the sigmoidal function is proposed for the sign(·) and Heaviside functions. Since the accuracy of this approximation is highly dependent on the size of the sigmoidal parameter and the use of MATLAB for simulation to handle sign(·) function in this study, an approximation for the Heaviside functions is proposed as:

(6) $$H_{b}\left(I_{bus}\right)=\{\begin{array}{c} 1,\;\;I_{bus}>0\\ 0,\;\;I_{bus}\leq 0 \end{array}=floor\left(\frac{sign\left(I_{bus}\right)+1}{2}\right)\;H_{f}\left(V\right)=\{\begin{array}{c} 0,\;\;V>0\\ 1,\;\;V\leq 0 \end{array}=-sign\left(sign\left(V\right)-1\right)\;H_{s}\left(\left|\omega_{m}\right|-\omega_{s}\right)=\{\begin{array}{c} 1,\;\;abs\left(\omega_{m}\right)-\omega_{s}\geq 0\\ 0,\;\;abs\left(\omega_{m}\right)-\omega_{s}<0 \end{array}=round\left(\left(sign\left(abs\left(\omega_{m}\right)-\omega_{s}\right)+1\right)/2\right)$$

Therefore, the proposed $H_{b}$, $H_{f},$ and $H_{s}$ are no longer dependent on the size of the sigmoid parameter. 

### 2.2. Degradation Model

Considering lubricant evaporation and creeping due to temperature gradient, Jin et al. [19] suggest an exponential relationship between the lubricant’s temperature and the loss rate of lubricant as:

(7) $$\beta \left(t\right)=\beta_{0}e^{-b/T_{lub}\left(t\right)}+w_{k}$$

where $\beta_{0}$ and b indicate model parameters to be estimated, $T_{lub}\left(t\right)$ is the temperature of the lubricant at time t and $w_{k}$ is the process noise with the variance of $\sigma_{B}^{2}$. Assuming the effect of the temperature is immediate, and the damage incurred accumulates over time, the degradation X(t) at time t can be written as:

(8) $$X\left(t|T\left(\tau \right),0\leq \tau \leq t\right)=\beta_{0}\overset{t}{\underset{0}{\int }}e^{-b/T_{lub}\left(\tau \right)}d\tau +B\left(t\right)$$

where B(t) represents the Wiener process with the variance of $\sigma_{B}^{2}$. Since the estimation of the $T_{lub}$ and the amount of the injected lubricant is periodic, the degradation model can be presented in the discrete format as:

(9) X(ti)≈β0∑j=1tie−bTjΔ(ti)+B(ti)

This concludes the introduction of the degradation model in this study. In the next section, some background on the estimation theory will be provided for the state and parameter estimations in this study.

### 2.3. Particle Filter

The particle filter is a recursive Bayesian estimation technique based on the Monte Carlo simulation [27]. This method can approximate the posterior PDF of the state by sequentially selecting random samples (particles) and continuously adjusting their weights and positions according to new measurements.

To illustrate how PF works, a general nonlinear system can be written as:

(10) $$\{\begin{array}{l} x_{k}=f\left(x_{k-1},w_{k-1}\right)\\ y_{k}=h\left(x_{k},u_{k},\varepsilon_{k}\right) \end{array}$$

where $x_{k}\in {\mathbb{R}}^{n}$ and $y_{k}\in {\mathbb{R}}^{m}$ represent the unobservable state and measurement, respectively. $w_{k-1}$ and $\varepsilon_{k}$ are process and measurement noises, f(·) and h(·) indicate state transition and measurement equations, respectively, and $u_{k}$ is the known input of the system.

The PF process consists of two steps: (i) the prediction step and (ii) the update step. In the prediction step, base000000000d on the posterior PDF $p\left(x_{k-1}|y_{1:k-1}\right)$, the prior PDF at cycle k can be calculated as:

(11) $$p\left(x_{k}|y_{1:k}\right)=\int p\left(x_{k}|x_{k-1}\right)p\left(x_{k-1}|y_{1:k-1}\right)dx_{k-1}$$

where $y_{1:k}$ is $y_{1:k}=\left[y_{1},y_{2},\cdots ,y_{k}\right]$ and $p\left(x_{k}|x_{k-1}\right)$ denotes one-step transition probability. Using the Bayes’ rule and measurement at cycle k, the posterior PDF is updated as:

(12) $$p\left(x_{k}|y_{1:k}\right)=\frac{p\left(y_{k}|x_{k}\right)p\left(x_{k}|y_{1:k-1}\right)}{\int p\left(y_{k}|x_{k}\right)p\left(x_{k}|y_{1:k-1}\right)dx_{k-1}}$$

where $p\left(x_{k}|y_{1:k}\right)$ is the likelihood function. Consecutive calculations of Equations (11) and (12) will form a recursive Bayesian estimation. A Monte Carlo simulation method is used to estimate particle size as high-dimensional integrals, making it difficult to calculate PDFs analytically:

(13) $$p\left(x_{k}|y_{1:k}\right)=\overset{N}{\underset{i=1}{\sum }}w_{k}^{i}\delta \left(x_{k}^{i}-x_{k}\right)$$

where $x_{K}^{i}\;\left(i=1,\cdots ,N\right)$ are particles sampled from the importance function, δ(·) and N are the Dirac function and the number of particles, respectively. $w_{k}^{i}$ represent the corresponding weight calculated as:

(14) $$\;{\tilde{w}}_{k}^{i}=w_{k-1}^{i}\left[p\left(y_{k}|x_{k}^{i}\right)p\left(x_{k}^{i}|x_{k-1}^{i}\right)/q\left(x_{k}^{i}|x_{1:k}^{i},y_{1:k}\right)\right]\;;\;\;\;\;\;\;\;\;\;\;\;w_{k}^{i}={\tilde{w}}_{k}^{i}/\left(\overset{N}{\underset{j=1}{\sum }}\;{\tilde{w}}_{k}^{j}\right)$$

where $q\left(x_{k}^{i}|x_{1:k}^{i},y_{1:k}\right)$ determines the importance function. The transition probability represents an importance function in the standard form of PF $\left(q\left(x_{k}^{i}|x_{1:k}^{i},y_{1:k}\right)=p\left(x_{k}^{i}|x_{k-1}^{i}\right)\right)$, so the transition equation of weights can be simplified as:

(15) $$\;{\tilde{w}}_{k}^{i}=w_{k-1}^{i}p\left(y_{k}|x_{k}^{i}\right);\;\;\;\;\;\;\;\;\;\;\;\;\;w_{k}^{i}=\frac{\;{\tilde{w}}_{k}^{i}}{\sum_{j=1}^{N}\;{\tilde{w}}_{k}^{j}}$$

Based on the obtained $\;{\tilde{w}}_{k}^{i}$, the predicted state ($\hat{x}$) can be written as:

(16) $$\hat{x}=\overset{N}{\underset{j=1}{\sum }}\;w_{k}^{j}x_{k}^{i}$$

**Figure 4:**
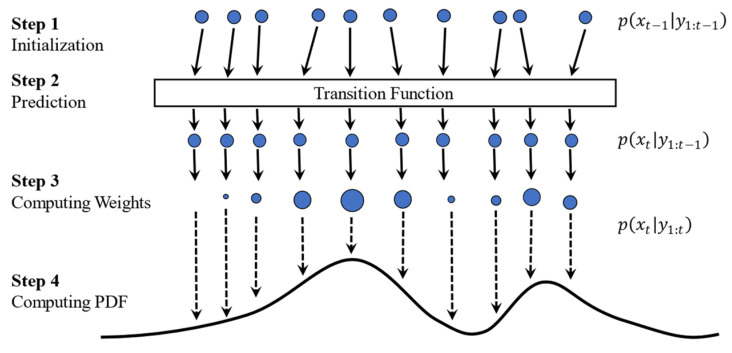
Steps associated with a Particle Filter approach. (1) Particle initialization; (2) prediction; (3) computing weights; and (4) computing PDF. Steps 3–4 are collectively known as the update phase.

Figure 4 shows the steps for the PF process. PF starts with a uniform distribution for the particles, i.e., all particles have the same size ($w_{k}^{i}=\frac{1}{N}$). After one transition step, particles are evaluated using the updated measurement ($p(y_{k}|x_{k}^{i})$), and the result forms a probability density function represented with a curve. The particles’ weight is then computed based on Equation (15), which is presented by the size of the particles. As shown in Figure 4, some particles have a large diameter (i.e., weight), others are smaller, and some have disappeared, meaning their weight is negligible.

The original formulation of the particle filter does not include a resampling stage, which can impose some issues on the filter’s performance. Hence, in the next section, resampling is discussed.

#### 2.3.1. Resampling

One issue with particle filtering is that after several iterations of particle propagation, the weight distribution among the particles becomes skewed, with few particles having a significant weight and most particles having very little weight. Resampling can address this degeneracy, but it may also lead to sample impoverishment, where only a small number of particles have significant weights while the majority of particles with low weights are discarded. To address this trade-off, resampling can be applied at predetermined intervals only when the variance of the non-normalized weights exceeds a certain threshold, indicating sample degeneracy. A method for determining the effective sample size ($N_{eff}$) is using [27,28]:

(17)  Neff≈1∑i=1N(wi2)

**Figure 5:**
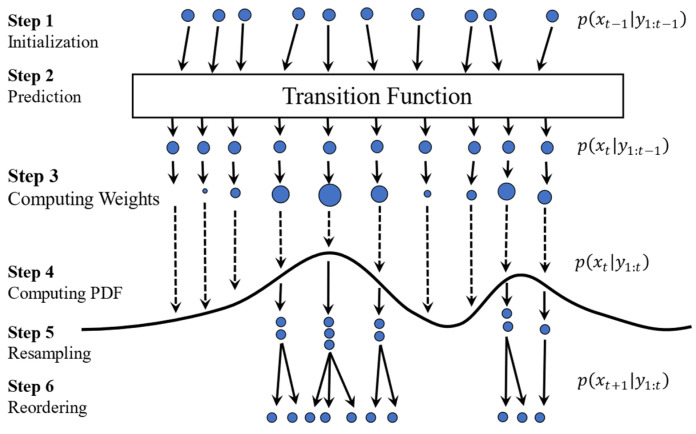
Steps associated with a Particle Filter approach with resampling. (1) Particle initialization (2) prediction; (3) computing weights; (4) computing PDF; (5) resampling; and (6) reordering.

Resampling only happens when a certain threshold ($N_{th}$) is reached. To renormalize the distribution, the resampling step duplicates the particles with large weights and eliminates those with small weights. The weight of each renormalized particle will then be set to 1/N [27]. Figure 5 shows the steps of PF when it benefits from the resampling step. At the resampling stage, particles with higher weights are broken into multiples, whereas particles with negligible weights are removed. Once resampling is performed, all the resampled particles get the same size because all weights are reset to 1/N as described in the initialization step. Notice that after the resampling step, the number of particles remains the same (N) but their distribution is different.

Although resampling can help renormalize the samples, performing it on each step due to a poor choice of $N_{eff}$ highlights the effect of abrupt noises and leads to confusion of PF for tracking. Section 3.1.1 further discusses this issue and how the proposed methodology can address it.

## 3. Methodology

In this section, the proposed methodology is detailed using the material provided in the earlier sections along with the enhancements put forward in this study.

In this study, as noted in the Introduction section and shown in Figure 2, a three-step prognosis approach for RUL estimation of RWs based on the lubrication consumption of the bearing unit is proposed in three steps as follows:

### 3.1. Step 1—Lubricant’s Temperature Estimation

Direct measurement of the lubricant’s temperature under the operating condition is generally not practical. Hence, in the first step of this approach, the lubricant’s temperature in the RW is estimated using its angular velocity ($\omega_{m}$) and motor current ($I_{m}$) as measurements and a high-fidelity mathematical model of the Ithaco RW as presented in Figure 3 and Equations (1)–(6) with model parameters in Table 2.

As mentioned in Section 2.3.1, although resampling in the formulation of PF can help renormalize the samples, it can be computationally expensive. One of the approaches to remedying this challenge is to use adaptive sampling; hence, a new adaptive sampling approach is proposed here to speed up the PF estimations, as detailed in the next section.

#### 3.1.1. Proposed Adaptive Resampling

In the adaptation proposed in this study, when the $p\left(y_{k}|x_{k}^{i}\right)$ in Equation (15) does not satisfy a certain threshold, the resampling step, despite its common purpose, spreads the particles in a broader range. For triggering this adaptation, the average of the maximum value of $p\left(y_{k}|x_{k}^{i}\right)$ in the interval of two triggered resampling steps is compared with a threshold ($p_{eff}$). This way of triggering the resampling stage prevents the sudden effect of abrupt noises and impacts when it senses the confusion of particles in an interval. In the case of abrupt changes, the combination of PF and adaptive resampling enables faster tracking of estimated states.

#### 3.1.2. Proposed Adaptive Sample Improvement

Since the f(.) and h(.) can both be nonlinear, the distribution of $p\left(y_{k}|x_{k}^{i}\right)$ can also be a function of other variable inputs ($u_{k}$) of the model. If $p\left(y_{k}|x_{k}^{i}\right)$ has an acceptable distribution, the difference between ${\tilde{w}}_{k}^{i}$ will increase gradually, and particles with higher probability will get highlighted. In the case that the $x_{k}$ has a low contribution to changing $y_{k}$, the ${\tilde{w}}_{k}^{i}$ would be distributed uniformly. The uniform distribution of ${\tilde{w}}_{k}^{i}$ leads to an inefficient resampling step. To overcome this issue, despite the PF, the importance function $q\left(x_{k}^{i}|x_{1:k}^{i},y_{1:k}\right)$ in Equation (15) can be replaced by:

(18) $$q\left(x_{k}^{i}|x_{1:k}^{i},y_{1:k}\right)=p\left(x_{k}^{i}|x_{k-1}^{i}\right)\gamma \left(\omega_{k-1}^{i},p\left(x_{k}^{i}|x_{k-1}^{i}\right),u_{k},N\right)$$

Assuming γ(.) is equal to:

(19) $$\gamma \left({\tilde{w}}_{k-1}^{i},p\left(x_{k}^{i}|x_{k-1}^{i}\right),u_{k},N\right)=w_{k-1}^{i}p\left(y_{k}|x_{k}^{i}\right)/\left[1-1/exp\left[c_{1}\left(u_{k}\right)\left[\beta -sign\left(\beta \right)c_{2}\right]\right]\right]\;\;\;\beta =\frac{1}{N}-w_{k-1}^{i}p\left(y_{k}|x_{k}^{i}\right)$$

where $c_{1}\left(u_{k}\right)$ is a function of known input variables of the system and $c_{2}$ is a constant, Equation (15) can be rewritten as:

(20) $$\;{\tilde{w}}_{k}^{i}=1-1/exp\left[c_{1}\left(u_{k}\right)\left[\beta -sign\left(\beta \right)c_{2}\right]\right];\;\;\;\;\;\;\beta =\frac{1}{N}-w_{k-1}^{i}p\left(y_{k}|x_{k}^{i}\right)$$

The right-hand side of Equation (20) shows an adapted logistic function with a maximum value of 1, and its growth rate is a function of $u_{k}$. The proposed mutation of ${\tilde{\omega }}_{k}^{i}$ can magnify the contribution of $x_{k}^{i}$ on $y_{k}$, which enhances the efficiency of the resampling step and prevents confusion in the filter.

**Figure 6:**
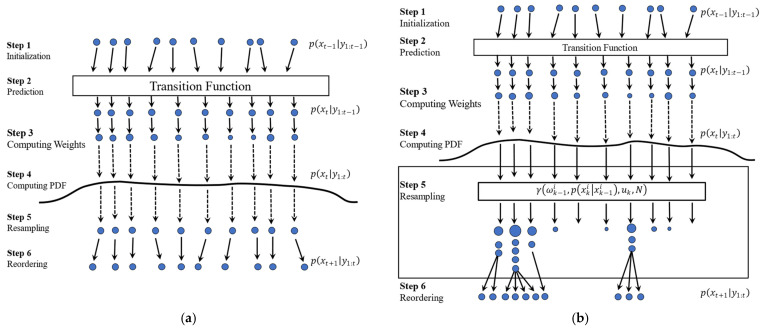
Comparison the steps associated with the (**a**) Particle Filter approach and the (**b**) Adaptive Particle Filter approach. (1) Particle initialization; (2) prediction; (3) computing weights; (4) computing PDF; (5) resampling; and (6) reordering.

Figure 6 compares the steps of APF proposed here, which benefit from the adaptive sample improvement step and classic PF discussed in Section 2.3. As can be seen, since the probability diagram has a uniform shape, the change in the weight of particles is not considerable enough to be recognized by the classic resampling step, as shown in Figure 6a. The adaptive sample improvement step, on the other hand, can distinguish this difference via the amplifying impact of Equation (19), as shown in Figure 6b.

Once the estimation is complete, the next step is to use the obtained information and propagate it in time to obtain the RUL, which is discussed next.

#### 3.1.3. Proposed Multi-Step Online Parameter Estimation

The proposed parameter estimation method has been developed to address the estimation confusion error resulting in cases where the number of measurements is less than the number of to-be-estimated parameters.

The general parameter estimation problem in a nonlinear system can be written as:

(21) $$\{\begin{array}{l} \theta_{k}=\theta_{k-1}+\alpha_{k}\\ y_{k}=h\left(\theta_{k},u_{k}\right) \end{array}$$

where $\theta_{k}$, $\alpha_{k}$, h(.), and $u_{k}$ are the parameter vector of the model, fault parameter vector, transition function, and the input vector of the model, respectively.

Assuming the parameter vector of the model consists of n parameter as:

(22) $$\theta_{k}=\left\{\theta_{k}^{i}\right\}\;;\;\;\;\;\;\;\;\;\;\;\;\;\;\;\;\;\;\;\;i=1,\ldots ,\;n$$

the multi-step parameter estimation is applicable if $\theta_{k}^{i}$ can be singled as:

(23) $$y_{k}=h^{i}\left(\theta_{k}^{i},u_{k}\right)\;y_{k-j};\;\;\;\;\;\;\;\;\;\;\;\;\;\;\;\;\;\;\;\;\;\;\;\;\{\begin{array}{c} \;i\in \left\{1,\cdots ,n\right\}\\ j\in \;\left\{1:k-1\right\} \end{array}\;\;\;\;\;\;\;$$

Having Equation (23), the sub-nonlinear system equation for $\theta_{k}^{i}$ can be presented as:

(24) $$\{\begin{array}{c} \theta_{k}^{i}=\theta_{k-1}^{i}+\alpha_{k}^{i}\\ \frac{y_{k}}{y_{k-j}}=h^{i}\left(\theta_{k}^{i},u_{k}\right) \end{array}$$

**Figure 7:**
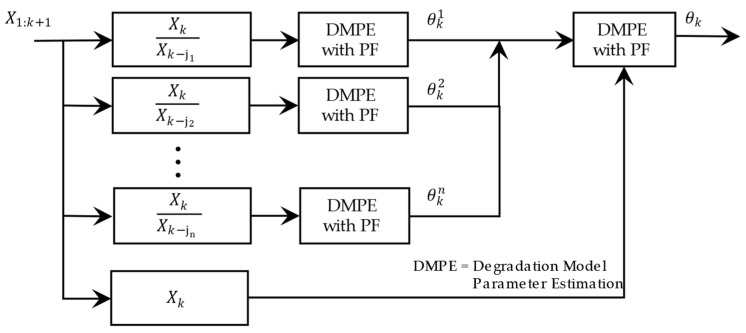
Flow of multi-step online parameter estimation.

As can be seen with the estimation of $\theta_{k}^{i}$ using the obtained sub-nonlinear system, PF is independent of other model parameters and resolves the confusion in the estimation. Figure 7 shows the flow of the proposed multi-step parameter estimation method.

### 3.2. Step 2—Degradation Model’s Estimation

In the second step, an online parameter tracking of the lubricant consumption model, as well as the degradation model, is conducted using the estimated lubricant’s temperature and the measured amount of injected lubricant.

### 3.3. Step 3—Remaining Useful Life Estimation

The last step is to predict the RUL of RW based on two scenarios of failure: (1) The cumulative consumption of lubricant reaches the amount stored in the system. (2) Deficiencies in lubrication injection and supplemental lubrication systems lead to bearing unit dry-out. In the first scenario, the fault occurs during normal operating conditions and will be accelerated by lubrication bleeding or RW heating. However, a fault in the supplementary lubrication system causes an unbalanced lubrication injection in the second scenario (see Figure 2).

#### Prediction of RUL

In general, the *RUL* of a system at time k can be defined as:

(25) $$\;RUL_{k}=t_{end}-t_{k}$$

where $t_{k}$ is the time k and $t_{end}$ is failure time which is defined as:

(26) $$\;t_{end}=t\left({\hat{X}}_{t}=X_{th}|{\hat{\theta }}_{k}\right)$$

In which ${\hat{\theta }}_{k}$, ${\hat{X}}_{t}$, and $X_{th}$ stand for the estimated parameters of the degradation model, the predicted state of the system in future time t based on the estimated parameter at time k (${\hat{\theta }}_{k}$), and the threshold which defines the system’s failure, respectively.

In the next section, the preliminary material and the proposed methodology are put together to show results and discussions for a case study on a RW’s remaining useful life estimation based on its residual lubricant level for the bearing unit and its impact on the unit’s life expectancy.

## 4. Results and Discussion

In this study, the RUL of RWs, based on the residual lubricant level for the bearing unit, is studied under two incipient fault scenarios to evaluate the proposed method’s effectiveness:

(1) **Normal or excessive lubricant loss**. Under this scenario, the RW will operate until it runs out of lubricant. In this case, the cumulative loss of lubricant acts as the HI of the system, and the maximum amount of lubricant it can carry is the threshold;
(2) **Insufficient lubrication caused by a fault in the supplementary lubrication system**. This scenario results in bearings drying out while there is still lubricant in the supplementary lubrication system. For this scenario, HI is computed as the difference between the normal lubricant loss and the injection amount, and the failure threshold is the amount of lubrication loss the bearing unit can tolerate without additional lubrication.

**Figure 8:**
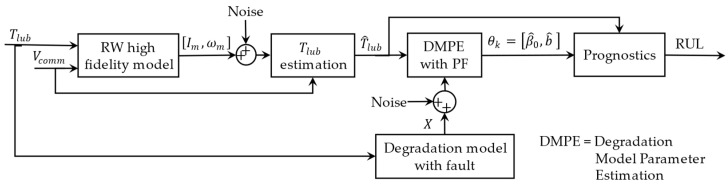
The flow of temperature estimation leads to online parameter estimation for the degradation model and prognostics.

The simulations were set up, as shown in Figure 8. The Runge–Kutta method (RK4), with a sampling interval of 0.05 s, was used in MATLAB for the numerical integration of the states. The simulation parameters are as listed in Table 2, while white noise with a standard deviation $\sigma_{n}$ was added to the nominal states to produce measured states.

### 4.1. Step 1—Lubricant’s Temperature Estimation

In this section, the performance of the first step of the proposed method is examined by following a temperature path introduced in Table 3. As shown in Figure 8, the proposed temperature path ($T_{lub}$) is fed to the RW’s model, and angular velocity and motor current ($\left[\omega_{m},\;I_{m}\right]$) are considered the measurements. After adding the white noise to the measured values, they are fed to the state estimation block with $V_{comm}$ as the system’s input.

The objective of the state estimation block is to estimate the temperature of the lubricant (${\hat{T}}_{lub}$) as the unmeasured state of the system using the available measurements, input variables, and the model of the RW. The nonlinear model of the system (Equation (10)) can be presented as follows:

(27) $$\{\begin{array}{c} x_{k}={\left(T_{lub}\right)}_{k}={\left(T_{lub}\right)}_{k-1}+w_{k-1}\\ y_{k}=\left[{\left(\omega_{m}\right)}_{k},\;{\left(I_{m}\right)}_{k}\right]=h\left({\left(T_{lub}\right)}_{k},{\left(V_{comm}\right)}_{k},\varepsilon_{k}\right) \end{array}$$

where h(.) represent the RW’s model.

**Figure 9:**
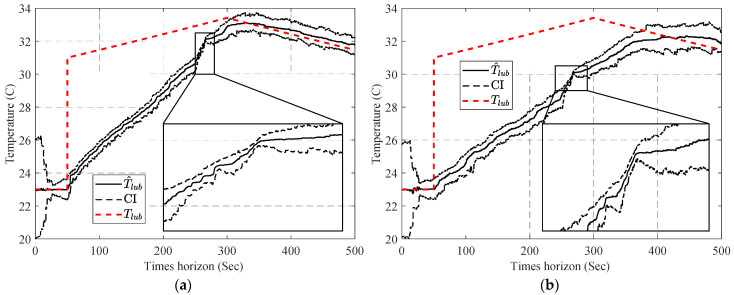
Result of the estimated $T_{lub}$, using PF: (**a**) low noise scenario and (**b**) high noise scenario.

Figure 9 shows the performance of PF in the estimation of $T_{lub}$ under low ($\sigma_{I}=3\times 10^{-2},\;\sigma_{\omega }=3\times 10^{-3}$) and high ($\sigma_{I}=3\times 10^{-2},\;\sigma_{\omega }=3\times 10^{-3}$) noise conditions. As it can be seen, the PF is able to accurately track the changes in $T_{lub}$, if these changes are not significant. In the case of abrupt changes, the PF convergence is no longer acceptable.

**Figure 10:**
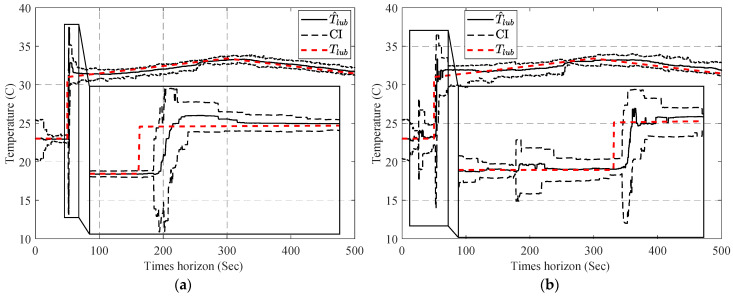
Result of the estimated $T_{lub}$, using a combination of PF and adaptive resampling: (**a**) low noise scenario and (**b**) high noise scenario.

To overcome this issue, an adaptation of resampling (explained in Section 3.1.1) is used in this study. Figure 10 shows the performance of the combination of PF and the adapted resampling step in the estimation of $T_{lub}$.

Results in Figure 10 show that adaption in the resampling step significantly improved the ability of the PF to trace sudden changes in $T_{lub}$; however, Figure 10b illustrates that this accuracy improvement comes with inaccuracy in the confidence interval. The source of this inaccuracy is the low contribution of temperature to changing the measured variables compared with the effect of measurement noise; this low contribution leads to an almost uniform distribution of $p\left(y_{k}|x_{k}^{i}\right)$ and inefficiency of the resampling step in narrowing down the confidence interval (CI) and consequently not satisfying the $N_{eff}$ (Equation (17)), which results in repeating the resampling step after any update step.

To address the inefficiency of the resampling step, the proposed adaptive sample improvement (explained in Section 3.1.2) is adjusted in a way that Equation (20) can be rewritten as:

(28) $$\;{\tilde{w}}_{k}^{i}=1-1/exp\left[-10^{10-V_{comm}}\times \left[\beta -0.6\times sign\left(\beta \right)\right]\right]\;\;\;\;\;\;\beta =\frac{1}{N}-w_{k-1}^{i}p\left(y_{k}|x_{k}^{i}\right)$$

**Figure 11:**
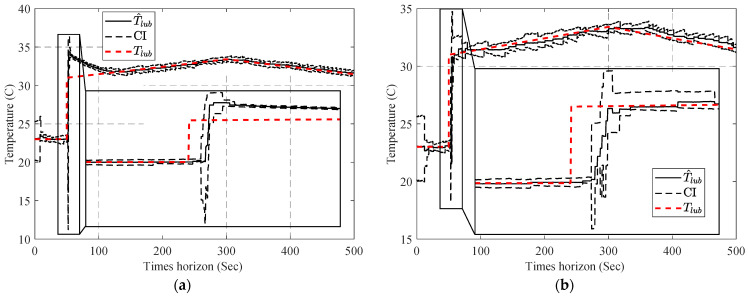
Result of the estimated $T_{lub}$, using APF: (**a**) low noise scenario and (**b**) high noise scenario.

This adjustment amplifies the differences in$\;{\tilde{\omega }}_{k}^{i}$ and enables the resampling step to narrow down the CI. Figure 11 shows the performance of the APF on the same experimental condition.

### 4.2. Step 2—Degradation Model’s Estimation

**Figure 12:**
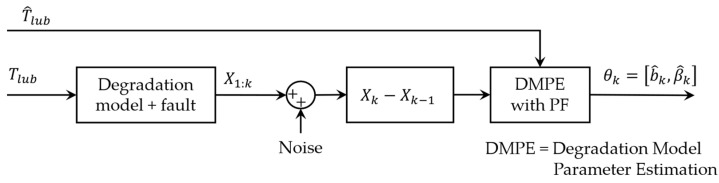
Flow of one-step online parameter estimation for degradation model.

As shown in Equation (8), the degradation model comes with two parameters ($\beta_{0}$ and b) that need to be estimated to predict the evolution of lubrication consumption. For this purpose, a long-term estimation of the lubricant’s temperature through PF and APF was simulated. The degradation model parameters were then estimated using a PF block fed by the estimated temperature of lubricant and simulated measurements of lubrication consumption. Figure 12 indicates the flow of online parameter estimation for the degradation model.

As it is impractical to measure lubrication consumption over 0.05 s, which is the time step necessary for estimating the temperature, *X* is calculated as the cumulative sum of lubrication consumption over 4 min using Equation (29).

(29) $$\;X_{k}=\overset{4800k}{\underset{j=1}{\sum }}\beta_{k}\;exp\left(\frac{b_{k}}{T_{j}}\right)\;\Delta t$$

where Δt is equal to 0.05 s. The nonlinear model of the degradation problem (Equation (22)) can be rewritten as:

(30) $$\{\begin{array}{l} \theta_{k}=\left[b_{k},\beta_{k}\right]=\left[b_{k-1},\beta_{k-1}\right]+\alpha_{k}\\ y_{k}=X_{k}-X_{k-1}=\overset{4800k}{\underset{j=4800\left(k-1\right)}{\sum }}\beta_{k}exp\left(\frac{b_{k}}{T_{j}}\right)\;\Delta t \end{array}$$

**Figure 13:**
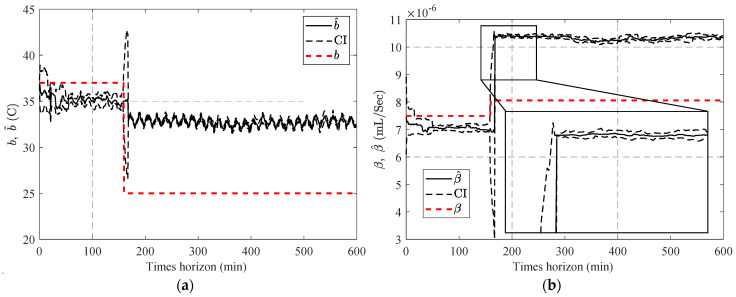
Result of the estimated (**a**) $\hat{b}$ and (**b**) $\hat{\beta }$ using the one-step online parameter estimation method.

**Figure 14:**
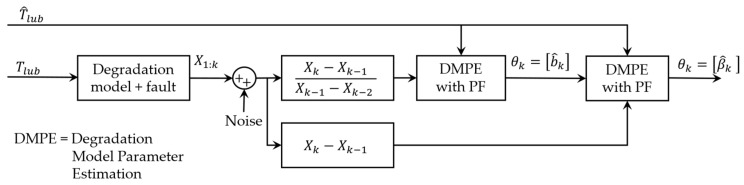
Flow of two-step online parameter estimation for degradation model.

**Figure 15:**
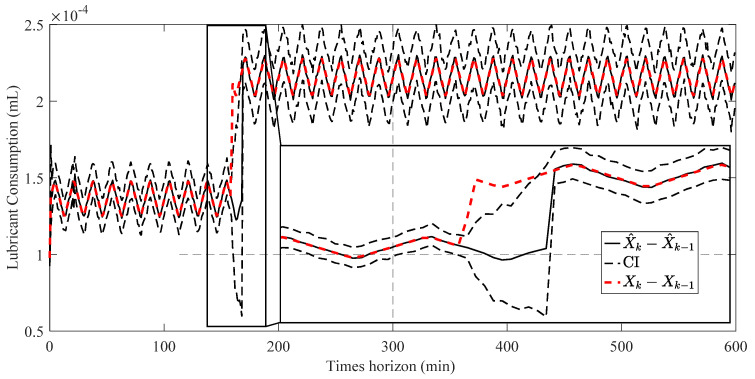
Result of the estimated lubrication consumption (${\hat{X}}_{k}-{\hat{X}}_{k-1}$) using the one-step online parameter estimation method.

Figure 13 compares the estimated parameters ($\hat{b},\hat{\beta }$) with their actual values. In order to address this issue, a two-step particle filter (Figure 14) is formed from the proposed multi-step online parameter estimation (explained in Section 3.1.3). As can be seen, estimated $\hat{b}$ and $\hat{\beta }$ cannot accurately follow their actual values even though the estimated lubrication consumption path shown in Figure 15 overlaps the actual one.

The first PF block uses the ratio of three consecutive measurements of lubrication consumption to estimate b using:

(31) $$\;\frac{X_{k}-X_{k-1}}{X_{k-1}-X_{k-2}\;}=exp\left(b_{k}\left(\frac{1}{T_{k-1}}-\frac{1}{T_{k}}\right)\right)$$

where $X_{k}$ and $T_{k}$ are the cumulative consumption of lubricant and the temperature of lubricant at time k. Applying the alternation presented in Equation (29) converts Equation (31) to:

(32) $$\;\frac{X_{k}-X_{k-1}}{X_{k-1}-X_{k-2}\;}=\sum_{j=4800\left(k-1\right)}^{4800k}\exp\left(-b/Tj\right)/\sum_{j=4800\left(k-2\right)}^{4800\left(k-1\right)}\exp\left(-b/Tj\right)$$

Using Equation (32), the sub-nonlinear system equation in Equation (24) can be written as:

(33) $$\{\begin{array}{l} \theta_{k}^{1}=b_{k}=b_{k-1}+\alpha_{k}^{1}\\ \frac{y_{k}}{y_{k-1}}=\frac{X_{k}-X_{k-1}}{X_{k-1}-X_{k-2}\;}=\overset{4800k}{\underset{j=4800\left(k-1\right)}{\sum }}\exp\left(-b/Tj\right)/\overset{4800\left(k-1\right)}{\underset{j=4800\left(k-2\right)}{\sum }}\exp\left(-b/Tj\right) \end{array}$$

(34) $$\{\begin{array}{c} \theta_{k}^{2}=\beta_{k}=\beta_{k-1}+\alpha_{k}^{2}\\ y_{k}=X_{k}-X_{k-1}=\overset{4800k}{\underset{j=4800\left(k-1\right)}{\sum }}\beta_{k}\;exp\left(\frac{{\hat{b}}_{k}}{T_{j}}\right)\;\Delta t \end{array}$$

**Figure 16:**
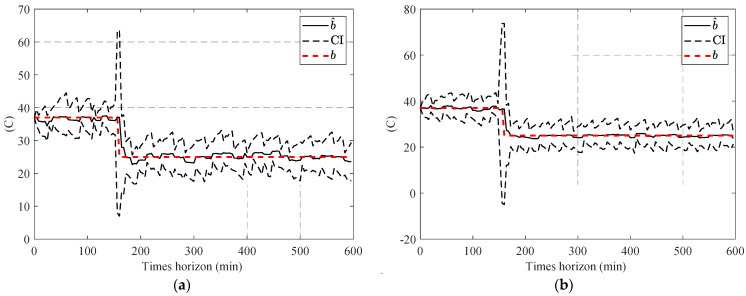
Result of the estimated $\hat{b}$ using the two-step method and estimated the lubricant’s temperature via: (**a**) PF and (**b**) APF.

Figure 16 compares the estimated $\hat{b}$ using the estimated lubricant’s temperature from Section 4.1.

**Figure 17:**
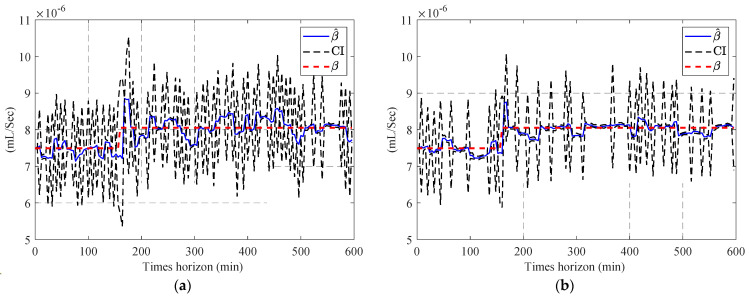
Result of the estimated $\hat{\beta }$ using the two-step method and estimated the lubricant’s temperature via: (**a**) PF and (**b**) APF.

Having the estimated $\hat{b}$, the second PF estimates the second parameter of the degradation model using Equation (34) as the model and cumulative consumption of the lubricant as the measurements. Figure 17 shows the estimated ${\hat{\beta }}_{k}$ for both estimated temperatures using PF and APF.

### 4.3. Step 3—Remaining Useful Life Estimation

This section evaluates the performance of the proposed three-step prognosis method using estimated degradation model parameters for both fault scenarios. As a result of the first fault scenario, the rate at which the lubricant is injected is greater than normal, resulting in a faster consumption of resources than expected. In contrast, the second fault scenario causes less lubrication injection, which cannot compensate for normal lubrication loss. The bearing unit dries out due to this scenario over time.

The failure time ($t_{end}$) from Equation (26) can be adjusted for the first fault scenarios as:

(35) $$\;t_{end}=t\left({\hat{X}}_{t}-X_{k}=X_{total}-X_{k}|\left[{\hat{b}}_{k},{\hat{\beta }}_{k}\right]\right)$$

where ${\hat{X}}_{t}$ and $X_{k}$ are estimated cumulative consumption of lubrication up to time t and k (current time), and $X_{total}$ represents the total lubrication that the system is carrying, respectively. However, the adjusted failure time for the second fault scenario should be considered as:

(36) $$\;t_{end}=t\left({\hat{X}}_{t}-X_{t}^{h}=X_{bearing}|\left[{\hat{b}}_{k},{\hat{\beta }}_{k}\right]\right)$$

where $X_{t}^{h}$ and $X_{bearing}$ are the cumulative consumption of lubrication up to time t in the healthy system and the maximum amount of lubrication that the bearing unit can lose without any effects on its capability, respectively. Table 4 lists the fault scenarios for evaluating the proposed method in RUL estimation.

**Figure 18:**
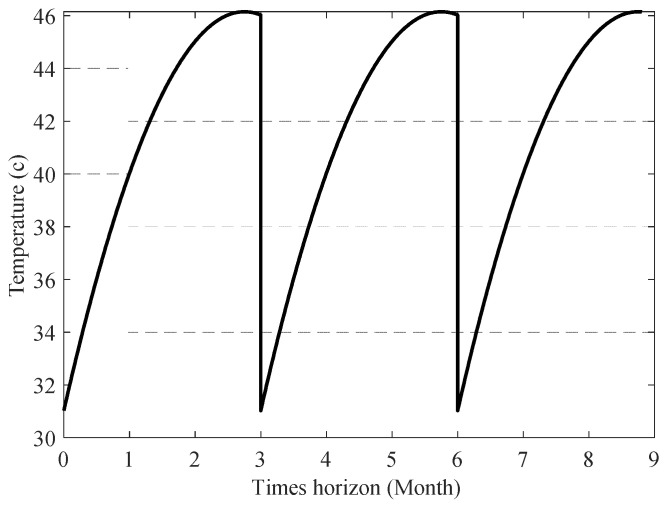
Assumed lubricant temperature (first fault scenario).

**Figure 19:**
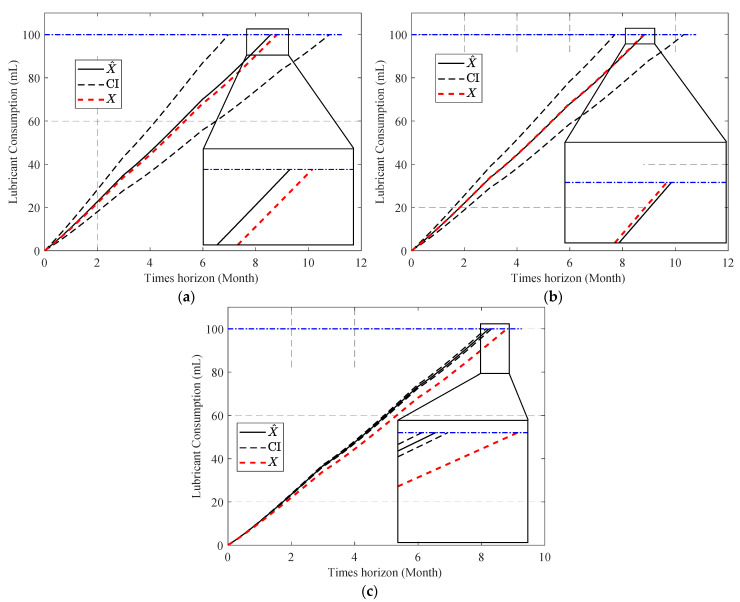
Failure prognosis results (first fault scenario): (**a**) using PF for temperature estimation and the two-step method for parameter estimation; (**b**) using APF for temperature estimation and the two-step method for parameter estimation; and (**c**) using APF for temperature estimation and the one-step method for parameter estimation.

**Figure 20:**
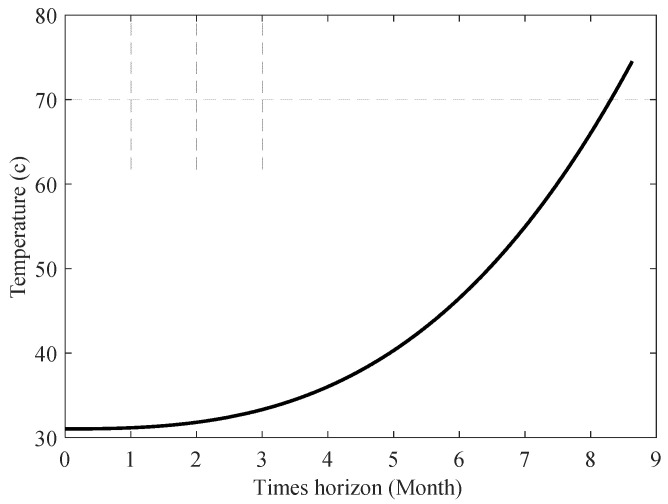
Assumed lubricant temperature (second fault scenario).

**Figure 21:**
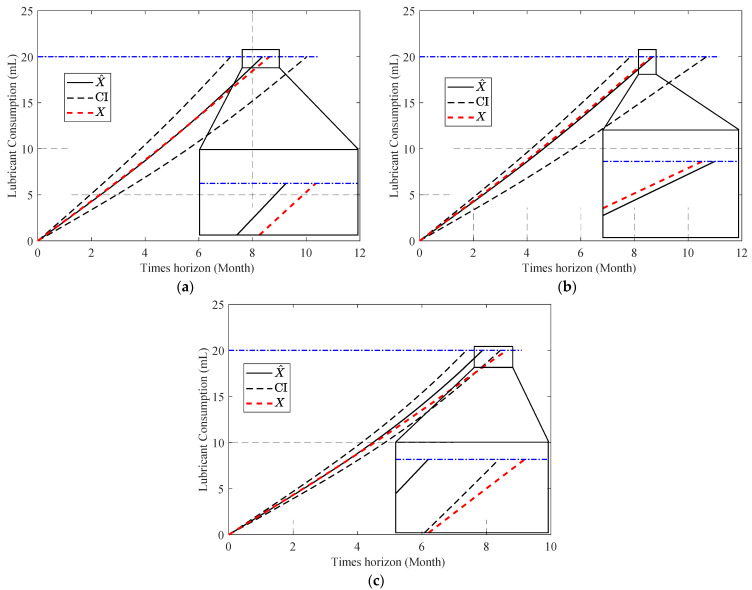
Failure prognosis results (second fault scenario): (**a**) using PF for temperature estimation and the two-step method for parameter estimation; (**b**) using APF for temperature estimation and the two-step method for parameter estimation; and (**c**) using APF for temperature estimation and the one-step method for parameter estimation.

Under the assumptions that the system is suffering from the first fault scenario, and the lubricant temperature (${\hat{T}}_{lub}$) changes periodically over three months, as displayed in Figure 18, Figure 19 shows the cumulative sum of lubrication consumption. Assuming the system is experiencing the second fault scenario, and the system’s temperature is gradually rising due to improper lubrication, as shown in Figure 20, Figure 21 shows the cumulative sum of normal lubrication consumption and injected lubricant difference.

### 4.4. Discussion

An enhanced estimation method APF based on the combination of PF and adaptive sample improvement (Section 3.1.2) has been proposed in this research to overcome the inefficiency of the resampling step due to the uniform distribution of particles. Based on the result shown in Figure 7, the proposed APF method performs better for lubricant temperature estimation than the combination of PF and adaptive resampling methods in higher noise cases. In low-noise cases, the APF results in more accuracy with a narrower CI; however, the low speed of recovery from confusion caused by voltage change causes less desirable results compared with the combination of PF and adaptive resampling. 

Table 5 compares the performance of combined PF and adaptive resampling with APF as estimation methods, using the RMSPE of the estimated lubricant temperature (${\hat{T}}_{lub}$). The outcome of the simulations can vary in each run due to the random nature of the data and particle generation. Therefore, a set of 20 simulations was conducted, and the average values of the whole set were reported under each measure. The lower RMSPE specifies better performance and more accurate estimates, meaning that in the higher measurement noise, APF performs better. Moreover, in both the APF and PF methods, the more particles, the better the accuracy, which comes at the price of computation.

In the next stage, one-step and two-step parameter estimation methods were applied for online parameter estimation of the degradation model based on the estimated temperature of the lubricant (Section 4.2). Based on the comparison of Figure 13, Figure 16 and Figure 17, the proposed two-step parameter estimation method is superior in terms of accuracy. Additionally, by analyzing Figure 17a,b, it can be seen that using the APF method to estimate the temperature of the lubricant will result in a more precise and less fluctuating $\hat{\beta }$ estimation.

Table 6 also shows the performance of combined methods of lubricant temperature and the degradation model’s parameter estimations in terms of RMSPE. Based on the presented values, the combination of APF and two-step PF results in the lowest RMSPE among all tested methodologies, indicating greater accuracy.

In the final step, the RUL of RW has been predicted under two fault scenarios and using the assumed temperature trend (Section 4.3). Comparing Figure 19 and Figure 21, the accuracy of using the two-step method in online parameter estimation of the degradation model leads to more precise RUL prediction. Table 7 compares the performance of the three tested methodologies for their accuracy in predicting RUL in both fault scenarios. Table 7 shows that the combined APF and one-step methods are not only not accurate enough to predict RUL, but the predicted result using that can also vary in a wide range, making it unreliable. As can be expected from the results shown in Table 6 and Table 7, the accuracy of using APF compared to PF in estimating lubricant temperature leads to a more accurate RUL prediction.

## 5. Conclusions

This study proposed a novel approach for estimating the RUL of satellites’ RW. The proposed methodology was evaluated on the RW remaining useful life estimation case study resulting from the amount of lubricant left in the RW’s bearing unit and potential faults in the supplementary lubrication system. The novelty was twofold: (1) the new adaptive resampling method for the particle filter detailed in Section 3.1.1 and 3.1.2; and (2) a new multi-step online parameter estimation method detailed in Section 3.1.3. The proposed approach consists of three steps: in the first step, using the RW’s angular velocity and motor current as available measurements, the APF was utilized to estimate the lubricant temperature in the bearing unit. In the second step, the amount of injected lubrication in the bearing, the estimated lubricant temperature, and the lubrication degradation model were fed to a two-step PF for online model parameter estimation. Finally, in the third step, RW’s RUL was predicted under two fault scenarios, including excessive lubrication loss and insufficient lubrication injection, to evaluate the performance of the proposed prognostic method. The results showed that the RUL was successfully predicted, with error rates ranging from ~0.1 to 4%. In conclusion, the proposed scheme can be considered a practical technique for long-term RUL estimation for deteriorating systems, including small satellites.

A combination of fault diagnostic and failure prognostic methods can ensure the validity of the system’s model parameter and the reliability of the prognostic process. A potential path for future works is to benefit from the proposed multi-step online parameter estimation method to monitor other parameters of the RW model, particularly the motor torque constant ($k_{t}$), which could improve the dependability and accuracy of lubricant estimated temperature. Further research can also focus on hybrid prognostic methods. Combining model-based approaches to estimate the current state of the system and data-driven methods to forecast the future path can enhance and narrow down the CI of the predicted RUL.

## Figures and Tables

**Table 1:** Samples of ACS and RW failure.

Year	Satellite	Cause of Failure	Ref
2001	BIRD	Experienced failures of 3 out 4 RWs, plus the gyroscope failure.	[3]
2001	Odin	Lost 1 RW mid-mission; a redundant RW allowed mission completion.	[3]
2006	FUSE	Four RWs failed over six years (2001–2007).	[6]
2008	Orbcomm 1–5 (5 units)	All satellites had problems with their RWs.	[3]
2009	SumbandilaSat	Lost Z-axis RW. Adaptive control algorithms allowed mission completion.	[3]
2013	Kepler space telescope	Experienced failures of two RW assemblies in 2012 and 2013.	[5,6]
2013	Dawn space probe	Experienced several failures of its reaction wheels.	[6]
2015	OCSD-A (AeroCube 7)	Attitude control failure.	[3]

**Table 2:** RW’s model parameters from the data in [25].

Parameter	Unit	Value
Input voltage ($V_{comm}$)	V	0–5
Drive Gain ($G_{d}$)	A/V	0.19
Driver Bandwidth (−3 dB) ($\omega_{d}$)	rad/sec	9
Motor Torque Constant ($K_{t}$)	N-m/A	0.029
Motor Back-EMF ($K_{e}$)	V/rad/sec	0.029
Speed limiter negative feedback gain ($K_{s}$)	V/rad/sec	95
Overspeed Threshold ($\omega_{s}$)	rad/sec	690
Coulomb friction ($\tau_{c}$)	N-m	0.002
Flywheel Inertia (J)	$N-m-sec^{2}$	0.0077
Number of Motor Poles (N)	–	36
Cogging Torque Amplitude (B)	N-m	0.22
Motor Torque Ripple Coefficient (C)	–	0
Input Resistance ($R_{IN}$)	Ω	2
Voltage Feedback Gain ($K_{f}$)	V/V	0.5
Torque Noise Angle Deviation ($\theta_{a}$)	rad	0.05
Torque Noise High Pass Filter Frequency ($\omega_{a}$)	rad/sec	0.2

**Table 3:** Validation scenario for the first-state estimation block.

Time Range (Sec)	$T_{lub}\;\left({}^{{}^{\circ}}C\right)$	$V_{comm}\;(\mathrm{Volt})$
t≤50	23	1
t=50	31	1
50<t<250	$T_{lub}\left(t-dt\right)\times \left[1/\exp\left(-3\times 10^{-4}\times t\right)\right]$	1
250≤t<300	$T_{lub}\left(t-dt\right)\times \left[1/\exp\left(-3\times 10^{-4}\times t\right)\right]$	3
t≥300	$T_{lub}\left(t-dt\right)\times \left[1/\exp\left(3\times 10^{-4}\times t\right)\right]$	3

**Table 4:** Fault scenarios for prognostics evaluation.

**First Scenario: Excessive Lubrication Loss**		
**Time Range (min)**	$b\;\left({}^{{}^{\circ}}C\right)$	β **(mL/sec)**
t≤159	37	$7.494\times 10^{-6}$
t>159	25	$8.054\times 10^{-6}$
**Second Scenario: Insufficient Lubrication Injection**		
**Time Range (min)**	$b\;\left({}^{{}^{\circ}}C\right)$	β **(mL/sec)**
t≤159	37	$7.494\times 10^{-6}$
t>159	49	$7.000\times 10^{-6}$

**Table 5:** Performance comparison for different types of estimation methods on $T_{lub}$ estimation.

	Low Noise $\sigma_{I}=3\times 10^{-2},\;\;\;\sigma_{\omega }=3\times 10^{-3}$		High Noise $\sigma_{I}=1.3\times 10^{-1},\;\;\;\sigma_{\omega }=3\times 10^{-3}$	
Number of Particles	RMSPE			
	PF & Adaptive Resampling	APF	PF & Adaptive Resampling	APF
50	2.359%	2.554%	3.596%	3.048%
100	2.216%	2.471%	3.118%	2.691%
150	2.012%	2.292%	2.745%	2.697%
200	1.983%	2.340%	2.742%	2.605%
250	2.015%	2.378%	2.735%	2.589%
300	1.935%	2.436%	2.707%	2.496%
350	1.919%	2.305%	2.748%	2.480%

**Table 6:** Performance comparison for degradation model estimation evaluation.

	First Fault Scenario		Second Fault Scenario	
Methodology	Average of RMSPE over 20 Simulation Runs			
	b	β	b	β
APF + Two-step PF	3.947%	2.051%	1.920%	2.709%
PF + Two-step PF	5.081%	3.586%	3.726%	4.707%
APF + one-step PF	136.999%	23.896%	66.172%	24.303%

**Table 7:** RUL estimation performance.

	First Fault Scenario			Second Fault Scenario		
Methodology	RUL Estimation Percentage Error over 20 Simulation Runs					
	Lower Bound	Upper Bound	Average	Lower Bound	Upper Bound	Average
APF + Two-step PF	0.137%	0.552%	0.346%	0.308%	4.322%	2.343%
PF + Two-step PF	0.868%	2.088%	1.578%	0.428%	4.780%	2.453%
APF + one-step PF	2.308%	8.561%	6.137%	6.675%	16.442%	11.602%

## Data Availability

Not applicable.
